# Influenza induces IL-8 and GM-CSF secretion by human alveolar epithelial cells through HGF/c-Met and TGF-α/EGFR signaling

**DOI:** 10.1152/ajplung.00290.2014

**Published:** 2015-04-10

**Authors:** Yoko Ito, Kelly Correll, Rachel L. Zemans, Christina C. Leslie, Robert C. Murphy, Robert J. Mason

**Affiliations:** ^1^Department of Medicine, National Jewish Health, Denver, Colorado;; ^2^Department of Medicine, University of Colorado, Aurora, Colorado;; ^3^Department of Pediatrics, National Jewish Health, Denver, Colorado;; ^4^Department of Pharmacology, University of Colorado, Aurora, Colorado

**Keywords:** alveolar epithelial cells, influenza, innate immune responses, hepatocyte growth factor/c-Met, transforming growth factor-α/epidermal growth factor receptor

## Abstract

The most severe complication of influenza is viral pneumonia, which can lead to the acute respiratory distress syndrome. Alveolar epithelial cells (AECs) are the first cells that influenza virus encounters upon entering the alveolus. Infected epithelial cells produce cytokines that attract and activate neutrophils and macrophages, which in turn induce damage to the epithelial-endothelial barrier. Hepatocyte growth factor (HGF)/c-Met and transforming growth factor-α (TGF-α)/epidermal growth factor receptor (EGFR) are well known to regulate repair of damaged alveolar epithelium by stimulating cell migration and proliferation. Recently, TGF-α/EGFR signaling has also been shown to regulate innate immune responses in bronchial epithelial cells. However, little is known about whether HGF/c-Met signaling alters the innate immune responses and whether the innate immune responses in AECs are regulated by HGF/c-Met and TGF-α/EGFR. We hypothesized that HGF/c-Met and TGF-α/EGFR would regulate innate immune responses to influenza A virus infection in human AECs. We found that recombinant human HGF (rhHGF) and rhTGF-α stimulated primary human AECs to secrete IL-8 and granulocyte macrophage colony-stimulating factor (GM-CSF) strongly and IL-6 and monocyte chemotactic protein 1 moderately. Influenza infection stimulated the secretion of IL-8 and GM-CSF by AECs plated on rat-tail collagen through EGFR activation likely by TGF-α released from AECs and through c-Met activated by HGF secreted from lung fibroblasts. HGF secretion by fibroblasts was stimulated by AEC production of prostaglandin E_2_ during influenza infection. We conclude that HGF/c-Met and TGF-α/EGFR signaling enhances the innate immune responses by human AECs during influenza infections.

a major complication of influenza a virus (IAV) infection is viral pneumonia, which can lead to the acute respiratory distress syndrome (ARDS) (39). Alveolar type II (ATII) cells are the major targets for influenza infection in the gas-exchange portion of the human lung (49). Infected epithelial cells produce cytokines that attract and activate neutrophils and macrophages, and these leukocytes can further damage the epithelial-endothelial barrier. Neutrophils play an important role in innate immune defense by phagocytosing pathogens, preventing further invasion, and removing cellular debris, whereas excessive accumulation of activated neutrophils can cause bystander tissue damage (51). In human cases of ARDS, neutrophil concentrations in bronchoalveolar lavage fluid (BALF) are positively correlated with disease severity (31, 39). One of the most challenging issues is how to control the cytokine storm and neutrophil-driven inflammation during catastrophic viral pneumonia.

Epidermal growth factor receptor (EGFR) is one of the receptor tyrosine kinases (RTKs), which is well known to play an important role in the regeneration of the damaged epithelium (9). Recently, a new role of the EGFR pathway has been reported. In bronchial epithelial cell lines and in a limited number of studies with primary human bronchial epithelial cells, influenza virus induces shedding of EGFR ligands and activation of EGFR, which stimulates IL-8 secretion and inhibits IFN-γ-inducible protein of 10 (IP-10/CXCL10) and IFN-λ (IL-29). In influenza-infected mice, pretreatment with EGFR inhibitor results in decreased viral infection, decreased macrophage inflammatory protein (MIP)-2, a murine IL-8 homolog, secretion, and decreased neutrophil recruitment but increased IP-10 and IFN-λ secretion in influenza-infected mice (18, 23, 45). Another RTK, c-Met, and its ligand hepatocyte growth factor (HGF) are also well known to regulate repair of damaged epithelium by stimulating cell migration, proliferation, and survival (14, 17, 25, 27, 34). HGF levels in the lungs are increased in ARDS (34), and plasma concentrations of HGF are significantly increased in patients with severe influenza infection (3). However, little is known about the influence of HGF/c-Met in immune responses, and the effect of HGF/c-Met and EGFR on innate immune responses by human alveolar epithelial cells (AECs) has not been investigated. Therefore, we hypothesized that c-Met and EGFR signaling would induce cytokines/chemokines in primary human AECs during influenza infection. Identifying the pathways that regulate neutrophil chemoattractants by primary human AECs is important for understanding the pathogenesis of influenza-induced ARDS and may represent a viable approach for the treatment of severe ARDS attributable to influenza. In this report, we studied cytokine/chemokine induction by recombinant human HGF (rhHGF) and rhTGF-α in human AECs and activation of c-Met and EGFR signaling in AECs during influenza infection.

## MATERIALS AND METHODS

### 

#### Donor information.

To isolate human primary ATII cells and fibroblasts (FBs), we obtained human lungs from deidentified organ donors whose lungs were not suitable for transplantation and donated for medical research through the National Disease Research Interchange (Philadelphia, PA) and the International Institute for the Advancement of Medicine (Edison, NJ). The Committee for the Protection of Human Subjects at National Jewish Health deemed this research as nonhuman subject research. We selected donors with reasonable lung function with a Pa_O_2_/Fi_O_2_ ratio of >225, no history of clinical lung disease, a chest radiograph that indicated no infection, and a time on the ventilator of <5 days. The sex, age, race, and smoking history were variable and were not selection criteria.__

#### Isolation and culture of human ATII cells.

We modified the human ATII cell isolation method published by Fang et al. (6, 15, 46, 48). Briefly, the middle lobe was perfused, lavaged, and then instilled with elastase (Worthington, Lakewood, NJ) for 40 min at 37°C. The lung was minced, and the cells were isolated by filtration and partially purified by centrifugation on a discontinuous density gradient made of Optiprep (Accurate Chemical Scientific, Westbury, NY) with densities of 1.080 and 1.040 and by positive selection with MACS MicroBeads human CD326 (epithelial cell adhesion molecules) (Miltenyi Biotech, Bergisch Gladbach, Germany). The isolated cells were resuspended in DMEM supplemented with 10% FBS and 2 mM glutamine, 2.5 μg/ml amphotericin B, 100 μg/ml streptomycin, 100 μg/ml penicillin G (GIBCO, Life Technologies, Rockville, MD), and 10 μg/ml gentamicin (Sigma-Aldrich, St. Louis, MO). Cells were plated on 12-well cell culture plates (Costar, Corning, NY) that had been previously coated with rat-tail collagen (RTC) (in house) in DMEM with 10% FBS. The culture medium was changed to DMEM with 5% FBS on *day 2* and to DMEM with 1 mg/ml BSA on *day 3*. On *day 4*, the cells were stimulated with 50 ng/ml rhHGF, 10 ng/ml rhTGF-α, or IAV, Puerto Rico/8/1934 (PR8) strain (a gift from Dr. K. Hartshorn, Boston University, Boston, MA) (multiplicity of infection, MOI = 0.5), and cell lysates and culture medium were harvested at 24 h after each stimulation. To evaluate the effect of the TGF-α/EGFR pathway on IL-8 and granulocyte macrophage colony-stimulating factor (GM-CSF) secretion by AECs, dimethyl sulfoxide (DMSO, Sigma-Aldrich) (a vehicle control for AG1478) or 2.5 μM AG1478, an EGFR inhibitor (BioVision, Milpitas, CA) was added. These AEC culture conditions were used for most experiments, and the resulting phenotype of the AECs would be type I-like cells (48). However, additional studies were performed with culture conditions designed to maintain the type II cell phenotype. These cells were isolated by the IgG adherence method to remove macrophages and cultured on a mixture of RTC and Matrigel (BD Bioscience, San Jose, CA) in the presence of 1% charcoal-stripped FBS, keratinocyte growth factor (KGF), 8 Bromo-cyclic AMP, and dexamethasone (33).

#### Coculture system of AECs with lung FBs.

Human primary ATII cells were plated on RTC-coated 12-well Millicell inserts (pore size 1.0 μm, membrane surface area 1.1 cm^2^, 1.5 million cells/cm^2^) (EMD Millipore, Darmstadt, Germany) in DMEM with 10% FBS. The culture medium was changed to DMEM with 5% FBS on *day 2* and to DMEM with 1 mg/ml BSA on *day 3*. FBs were isolated from the same deidentified donor lungs as ATII cells described above. Human lung FBs were prepared by explanting minced human lungs into 100-mm tissue culture dishes. The medium consisted of DMEM supplemented with 10% FBS, 2 mM glutamine, 100 U/ml penicillin G, 100 μg/ml streptomycin, 2.5 μg/ml amphotericin B, and 10 μg/ml gentamicin. After ∼3 days, FBs grew out from the edge of the explants, the medium and remaining explant tissue fragments were removed, and the adherent cells were grown to 50% confluence. The FBs were then passaged, grown to near confluence, and frozen. These frozen cells were expanded once and then were used for all experiments (*passage 2*). We confirmed FBs with immunostaining of vimentin (+), thy 1 (CD90) (+), and cytokeratin (−). FBs were plated on 12-well plates (0.25 million/cm^2^) (BD Bioscience) in DMEM including 10% FBS and cultured at 37°C in 10% CO_2_ for 2 days. The cells were then washed and cultured for another day in DMEM without serum. The following day, AECs plated on 12-well inserts were infected by PR8, and 1 h after inoculation an insert with AECs was placed over a well of a 12-well plate with FBs. Both the apical (0.2 ml DMEM with 1 mg/ml BSA) and basal (1 ml DMEM with 1 mg/ml BSA) culture medium were harvested and combined at 48 h after infection. To evaluate the effect of HGF/c-Met pathway on IL-8 and GM-CSF secretion by AECs, DMSO (a vehicle control for PHA665752) and 500 nM PHA665752 (TOCRIS Bioscience, Bristol, UK) (a phospho-c-Met inhibitor) were added.

#### HGF secretion by lung FBs stimulated by conditioned medium from AECs.

Human primary ATII cells (1.5 million cells/cm^2^) were plated on six-well cell culture plates (BD Bioscience) that had been previously coated with RTC in DMEM with 10% FBS. The culture medium was changed to DMEM with 5% FBS on *day 2* and to 1 mg/ml BSA on *day 3*. On *day 4*, cells were stimulated by IAV, PR8 strain (MOI = 0.5) with or without 10 μM indomethacin (Sigma-Aldrich) or 10 μg/ml IL-1 receptor antagonist (IL-1Ra) (R&D Systems, Minneapolis, MN), and conditioned medium (CM) was harvested at 24 h after infection. To evaluate the effect of CM from AECs infected by PR8 on HGF secretion by lung FBs, the CM was added to lung FBs plated on 12-well cell culture plates, and the culture medium was harvested at 48 h to measure HGF protein concentration by ELISA.

#### Cytokine multiplex panel.

To evaluate cytokines/chemokines secreted by AECs, the cells were stimulated by rhHGF and rhTGF-α, and the CM was analyzed by Milliplex Magnetic Human Cytokines/Chemokines Panel-17 Plex (EMD Millipore) [eotaxin, granulocyte colony-stimulating factor (G-CSF), GM-CSF, IL-1α, IL-1β, IL-1Ra, IL-6, IL-8, IP-10, monocyte chemotactic protein-1 (MCP-1), MIP-1α, MIP-1β, regulated on activation, normal T cell expressed and secreted (RANTES), vascular endothelial growth factor (VEGF), FB growth factor 2 (FGF2), fractalkine, and growth-related oncogene protein (GRO)]. Protein concentrations were measured according to the instruction manual.

#### Neutrophil migration assay.

**Fig. 1.**
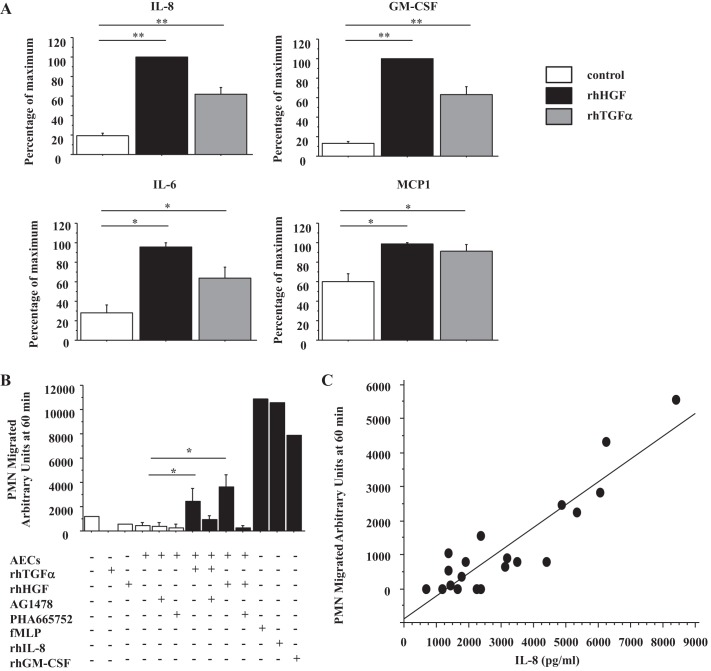
Recombinant human hepatocyte growth factor (rhHGF) and rh transforming growth factor (TGF)-α induce human alveolar epithelial cells (AECs) to secrete IL-8 and granulocyte macrophage colony-stimulating factor (GM-CSF) strongly, and IL-6 and monocyte chemotactic protein-1 (MCP-1) moderately, which attract neutrophils. *A*: IL-8, GM-CSF, IL-6, and MCP-1 protein concentrations in culture medium of human AECs stimulated by 50 ng/ml rhHGF or 10 ng/ml rhTGF-α for 24 h were measured by MILLPLEX; *n* = 6. Open bar, no growth factor; solid bar, rhHGF; shaded bar, rhTGF-α. The range of maximal stimulation: IL-8, 4,792.3–20,136.3 pg/ml; GM-CSF, 58.6–295.5 pg/ml; IL-6, 11.6–497.5 pg/ml; MCP-1, 26,184.5–104,165.1 pg/ml. *B*: degree of neutrophil migration was measured in vitro using conditioned medium AECs stimulated by rhTGF-α or rhHGF ±2.5 μM AG1478, ±500 nM PHA665752; *n* = 3. 1 μM *N*-Formyl-Met-Leu-Phe (fMLP), 10 ng/ml rhIL-8, and 10 ng/ml rhGM-CSF are positive controls. PMN, polymorphonuclear leukocytes. *C*: correlation diagram between IL-8 protein concentration in the same samples as *B* and the degree of neutrophil migration (*y* = −883.184 + 0.670 * *x*; *R*^2^ = 0.831) *P < 0.0001, **P < 0.05.

Human neutrophils were isolated from healthy donors as previously described (10) and in accordance with an approved Institutional Review Board protocol at National Jewish Health. Cells (8 × 10^6^/ml) were labeled with Calcein-AM (2.5 ìg/ml) in HBSS at 37°C for 15 min, washed once with HBSS, and resuspended (5 × 10^6^/ml) in Krebs-Ringers phosphate buffer with 0.2% dextrose (KRPD) with 1% heat-inactivated platelet-depleted poor plasma (HIPPP). Chemotaxis to CM from AECs, 50 ng/ml rhHGF, 10 ng/ml rhTGF-α, 10 ng/ml rhIL-8 (R&D Systems), 10 ng/ml rhGM-CSF (R&D Systems), 1 μM *N*-Formyl-Met-Leu-Phe (Sigma-Aldrich), or nondirected migration to KRPD with 1% HIPPP was assessed using modified Boyden chambers as previously described (2). Fluorescence in the bottom chamber was measured every 2 min for 120 min and reported in Fig. 1 as arbitrary units at 60 min (54).

#### Mass spectrometry for eicosanoids.

The culture medium was collected 24 h after PR8 infection (MOI = 0.5), centrifuged, and mixed with an equal volume of cold methanol for analysis. Mass spectrometry (MS) quantitation of eicosanoids [prostaglandin E2 (PGE_2_), thromboxane B2 (TXB_2_), leukotriene B4 (LTB4), LTE4, and hydroxyeicosatetraenoic acid (HETE)] in the CM was carried out as previously described (42).

#### HGF secretion by lung FBs stimulated by PGE_2_ and TXB_2_.

To determine which eicosanoid(s) (either PGE_2_ or TXB_2_, or both) stimulate(s) HGF secretion by lung FBs, 10^−9^ M PGE_2_ (Sigma-Aldrich) and/or 10^−7^-10^−5^ M TXB_2_ (Cayman Chemical, Ann Arbor, MN) were added to lung FBs plated on 12-well cell culture plates, and the culture medium was harvested at 48 h to measure HGF concentration by ELISA.

#### Real-time RT-PCR.

For real-time RT-PCR, the expression levels of genes were expressed as a ratio to the expression of the constitutive probe Cyclophilin B. The specific primers and probes for HGF, c-Met, TGF-α, and EGFR were purchased from Applied Biosystems (Foster City, CA).

#### ELISA assays and Western blotting.

To measure the concentration of HGF, TGF-α, GM-CSF, and IL-8 in the culture medium, we used a human HGF, TGF-α, GM-CSF (R&D Systems), and IL-8 ELISA (Elisa Tech, Aurora, CO) according to the manufacturers' instructions. For the Western blotting analysis, polyacrylamide gradient gels (8–16%; Invitrogen, Carlsbad, CA) were run in Tris-glycine buffer to separate the proteins. Protein loading was normalized to GAPDH. The primary antibodies to phosphorylated EGFR (Y1086) and EGFR antibody were from Abcam (Cambridge, MA).

#### Statistical analysis.

All data are presented as means ± SE. One-way ANOVA was used to compare the difference between two or more groups. The post hoc Bonferroni/Dunn test was used for multiple comparisons. However, because cytokine concentrations at baseline were variable from human to human, most of the data are shown as a percentage of maximum. The maximal levels observed in the individual experiments are stated in the figure legends. For the statistical analysis of percentage of maximum, repeated-measures ANOVA was used to compare the means between conditions. This statistical technique is used instead of the standard ANOVA because each sample is measured under several conditions, which means that some of the measurements are correlated because they come from the same sample. The use of repeated-measures ANOVA allows for the modeling of this correlation, which is necessary to have standard errors calculated correctly. All analyses were conducted in SAS 9.3, and statistical significance was set at *P* < 0.05.

## RESULTS

### 

#### rhHGF and rhTGF-α stimulate IL-8 and GM-CSF secretion by human AECs.

In preliminary experiments, we found that rhHGF and rhTGF-α stimulated IL-8 secretion by AECs. To investigate which additional cytokines/chemokines were induced by human AECs stimulated by rhHGF and rhTGF-α, 17 cytokines/chemokines in supernatants from AECs stimulated by rhHGF and rhTGF-α were measured by Milliplex Magnetic Human Cytokines/Chemokines Panel-17 Plex. IL-8 and GM-CSF were strongly upregulated (*P* < 0.0001), and MCP-1 and IL-6 were moderately upregulated (*P* < 0.05) by rhHGF and rhTGF-α (*n* = 6) (Fig. 1*A*). The maximal levels of IL-8 stimulated by HGF in these experiments ranged from 4.8 to 20.1 ng/ml. VEGF, fractalkine, and GRO were detected but not upregulated by rhHGF and rhTGF-α (data not shown). FGF2, eotaxin, G-CSF, IL-1α, IL-1β, IL-1Ra, IP-10, MIP-1α, and MIP-1β were detected at very low concentrations or not detectable (data not shown). As IL-8 (51) and GM-CSF (53) are both neutrophil chemoattractants, we evaluated whether AECs stimulated by rhHGF and rhTGF-α could accelerate neutrophil migration in vitro. rhHGF and rhTGF-α by themselves did not attract neutrophils, but CM from AECs stimulated by rhHGF and rhTGF-α enhanced neutrophil migration, which was abrogated by each specific receptor inhibitor (*n* = 3) (Fig. 1*B*). The level of IL-8 in the CM ranged from 0.6 to 8.4 ng/ml in these experiments. The degree of neutrophil migration correlated positively with the concentration of IL-8 in the CM (*y* = −883.184 + 0.670 * *x*; *R*^2^ = 0.831) (Fig. 1*C*).

Additionally, in a previous genechip study, we found that human AECs expressed seven potential EGFR ligands. Amphiregulin (AREG) and heparin-binding epidermal growth factor (HB-EGF) were expressed strongly, TGF-α moderately, and the others very weakly (47). However, rhAREG and rhHB-EGF did not stimulate IL-8 and GM-CSF secretion by human AECs (data not shown). In addition, neuregulin, a ligand of EGFR family ErbB3/ErbB2 or ErbB4/ErbB2, did not stimulate IL-8 secretion by the AECs.

#### TGF-α, EGFR, and c-Met are expressed in human AECs, but HGF is not.

**Fig. 2.**
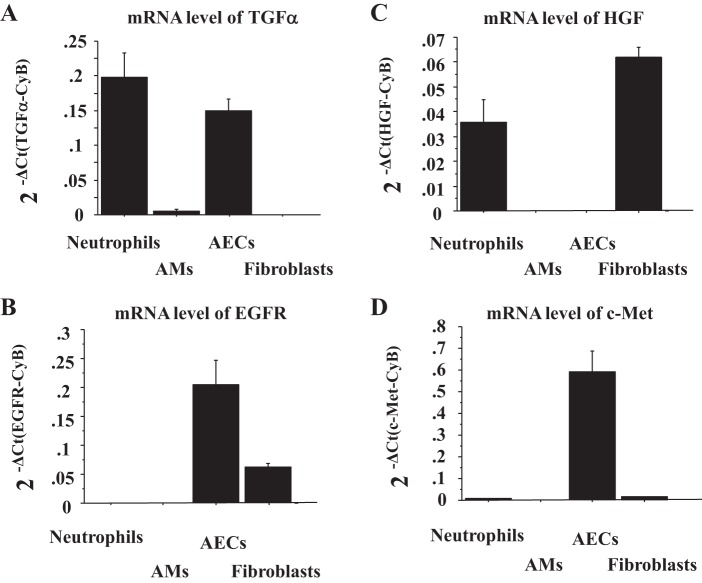
Human AECs express TGF-α, epidermal growth factor (EGFR), c-Met, but not HGF. The mRNA levels of TGF-α (*A*), EGFR (*B*), HGF (*C*), and c-Met (*D*) were measured by real-time PCR. These levels were normalized to the constitutive probe cyclophilin B (CyB). Values were means ± SE (*n* = 3 for each cell type). AMs, alveolar macrophages.

To examine which cell types expressed TGF-α, EGFR, HGF, and c-Met, we measured their mRNA levels in human neutrophils, alveolar macrophages (AMs), AECs, and FBs by RT-PCR. TGF-α was expressed by neutrophils and AECs (Fig. 2*A*), whereas its receptor EGFR was expressed by AECs and lung FBs (Fig. 2*B*) (*n* = 3). HGF was expressed by neutrophils and FBs but not by AECs (Fig. 2*C*), whereas its receptor c-Met was highly expressed by AECs (Fig. 2*D*) (*n* = 3). Therefore, in the human lung in vivo, HGF/c-Met signaling likely requires cell-cell communication between AECs and FBs or neutrophils.

#### IAV induces TGF-α secretion and activates EGFR, which stimulates IL-8 and GM-CSF secretion by AECs.

**Fig. 3.**
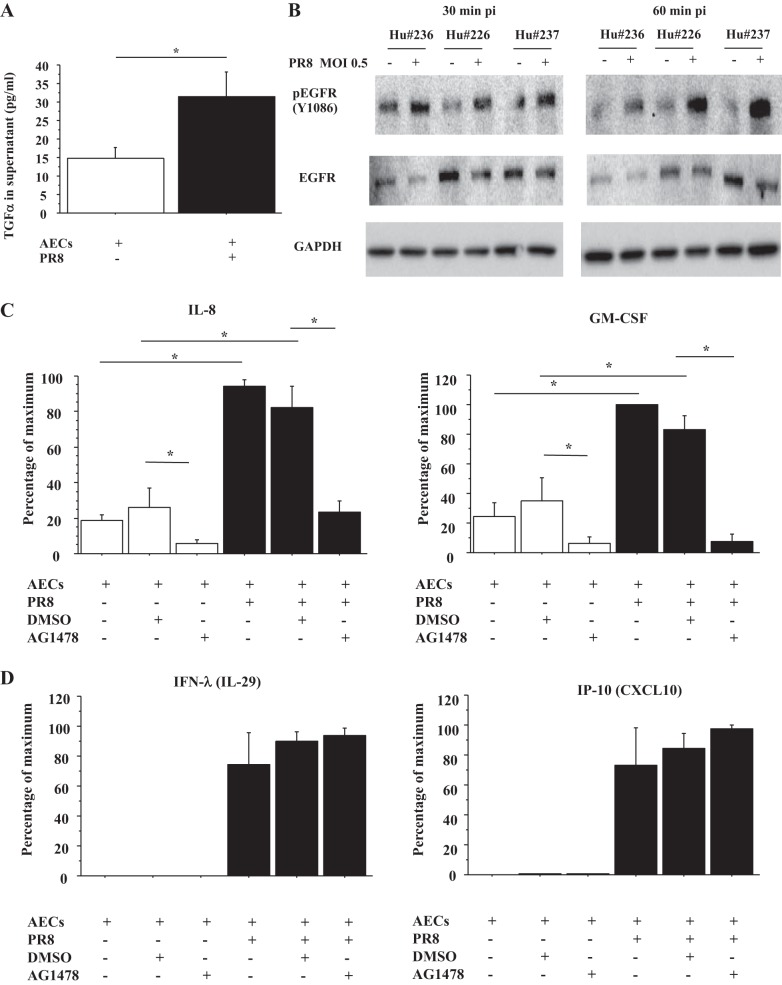
Influenza virus infection of AECs induces TGF-α secretion and activates EGFR, which stimulates secretion of IL-8 and GM-CSF by AECs. *A*: TGF-α concentration of culture medium from AECs with or without influenza A virus/Puerto Rico/8/1934 (PR8) infection (multiplicity of infection, MOI = 0.5) for 24 h was measured by ELISA. Values were means ± SE for 5 different human donors. *P < 0.05. *B*: human (Hu) AECs were infected by PR8 (MOI = 0.5). The AECs were harvested at 30 and 60 min after infection, and protein levels of phospho-EGFR and EGFR normalized by GAPDH were measured by immunoblotting. This blot is from 3 different human donors. pi, postinfection. *C*: IL-8 and GM-CSF concentration of culture medium of AECs with or without PR8 infection (MOI = 0.5) and with or without AG1478, an EGFR inhibitor, for 24 h was measured by ELISA (*n* = 5 for IL-8, *n* = 4 for GM-CSF, as 1 human sample was below limits of quantitation). DMSO was a vehicle control for AG1478. The range of maximal stimulation: IL-8, 1,250.4–7,089.6 pg/ml; GM-CSF, 6.9–95.0 pg/ml. *D*: IFN-λ (IL-29) and CXCL10 (IFN-γ-inducible protein of 10, IP-10) concentration of culture medium of AECs with or without PR8 infection (MOI = 0.5) and with or without AG1478 for 24 h was measured by ELISA (*n* = 3). DMSO was a vehicle control for AG1478. The range of maximal stimulation: IFN-λ, 753.6-5,933.4 pg/ml; IP-10, 1,174.8–5,278.9 pg/ml.

In bronchial epithelial cells, IAV infection leads to TGF-α shedding and activation of EGFR, which induces IL-8 secretion and suppresses IFN regulatory factor (IRF)-1-dependent IFN-λ (IL-29) and IP-10 (CXCL10) secretion (18, 45). Therefore, we examined whether IAV infection induced TGF-α secretion and altered IFN-λ and IP-10 secretion by human AECs. Human AECs were infected by PR8 (MOI = 0.5), and TGF-α concentration in the medium was measured by ELISA. As shown Fig. 3, PR8 infection induced TGF-α secretion by human AECs at 24 h after infection (*n* = 5) (Fig. 3*A*) and phosphorylation of EGFR (Y1086) at 30 and 60 min after infection (*n* = 3) (Fig. 3*B*). IL-8 secretion was induced by PR8 infection, which was inhibited by AG1478, an EGFR inhibitor (Fig. 3*C*). However, there was no alteration in influenza-induced IFN-λ and IP-10 secretion by AG1478 (Fig. 3*D*). As basal levels of IL-29 and CXCL10 were below level of detection in our ELISA, we could not detect the changes in the basal levels of IL-29 and CXCL10 by rhTGF-α, and rhTGF-α also did not alter IFN-λ and IP-10 secretion by influenza-infected AECs (data not shown). In addition, GM-CSF secretion by PR8 infection was also evaluated because, from the results of Milliplex, we knew that GM-CSF secretion by AECs was also stimulated by rhTGF-α (Fig. 1*A*). GM-CSF secretion was also stimulated by PR8 infection at 24 h after infection, which was inhibited by AG1478 (Fig. 3*C*). Interestingly, the baseline IL-8 and GM-CSF secretion by AECs was inhibited by AG1478 (Fig. 3*C*).

The previous observations were performed with influenza PR8 strain, which was propagated in chicken eggs. These results were confirmed with two strains of the 2009 pandemic H1N1 influenza (Cal 02/2009 and NY1682), which were grown in Madin-Darby canine kidney cells (55). The results with both viruses were similar to the results observed with PR8 (data not shown). To insure that the reduced cytokine production observed in the presence of the EGFR inhibitor AG1478 was not due to decreased viral infection, viral production was measured in the presence and absence of the inhibitor, and there was no decrease in viral production (*n* = 3). In addition, the amount of viral protein in the cells at 24 h after inoculation was the same with or without the EGFR inhibitor.

#### IAV infection stimulates IL-8 and GM-CSF secretion by AECs cocultured with lung FBs through c-Met signaling.

**Fig. 4.**
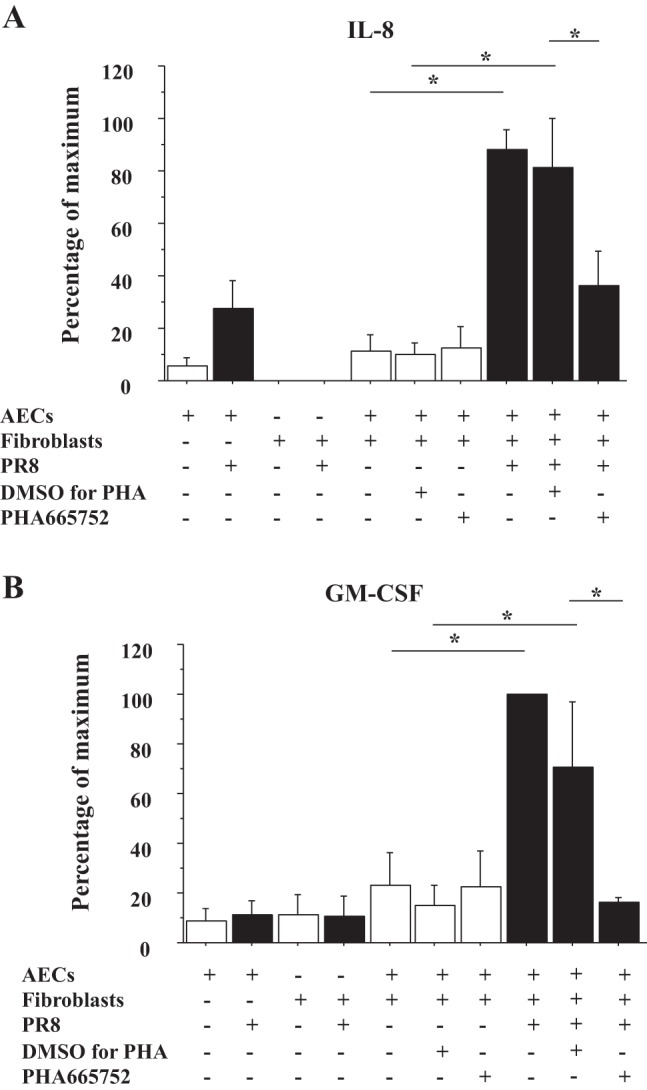
HGF/c-Met signaling induces IL-8 and GM-CSF secretion by AECs cocultured with lung fibroblasts (FBs) during influenza infection. AECs plated on the inserts were preincubated by PHA665752, a c-Met inhibitor, or vehicle control at 2 h before infection and then infected with PR8 (MOI = 0.5). At 1 h after infection, the AECs were placed over lung FBs plated on the plates to start coculture, and the culture medium was harvested at 48 h after starting the coculture. *A*: IL-8 concentration of these culture medium was measured by ELISA (*n* = 3). DMSO was a vehicle control for PHA665752. The range of maximal stimulation: IL-8, 4,188.1–26,022.0 pg/ml. *B*: GM-CSF concentration of same culture medium as *A* was measured by ELISA (*n* = 3). DMSO was a vehicle control for PHA665752. The range of maximal stimulation: GM-CSF, 44.0–953.5 pg/ml.

We found that rhHGF increased IL-8 and GM-CSF secretion by AECs (Fig. 1) and that in the human lung HGF was expressed by lung FBs not by AECs, whereas the HGF receptor c-Met was expressed by AECs (Fig. 2). Therefore, we hypothesized that HGF secreted by FBs would stimulate IL-8 and GM-CSF secretion by AECs during influenza infection. To examine this possibility, AECs plated on 12-well inserts were infected by PR8 (MOI = 0.5) with or without PHA665752, a c-Met inhibitor, placed over the FBs cultured on 12-well plates, and both apical and basal medium was harvested and combined at 48 h after infection to measure IL-8 and GM-CSF secretion. PR8 infection induced more IL-8 and GM-CSF secretion by AECs cocultured with FBs than AECs alone, which was inhibited by PHA665752 (*n* = 3) (Fig. 4, *A* and *B*).

#### PGE_2_ secreted by IAV-infected AECs stimulates HGF secretion by lung FBs.

**Fig. 5.**
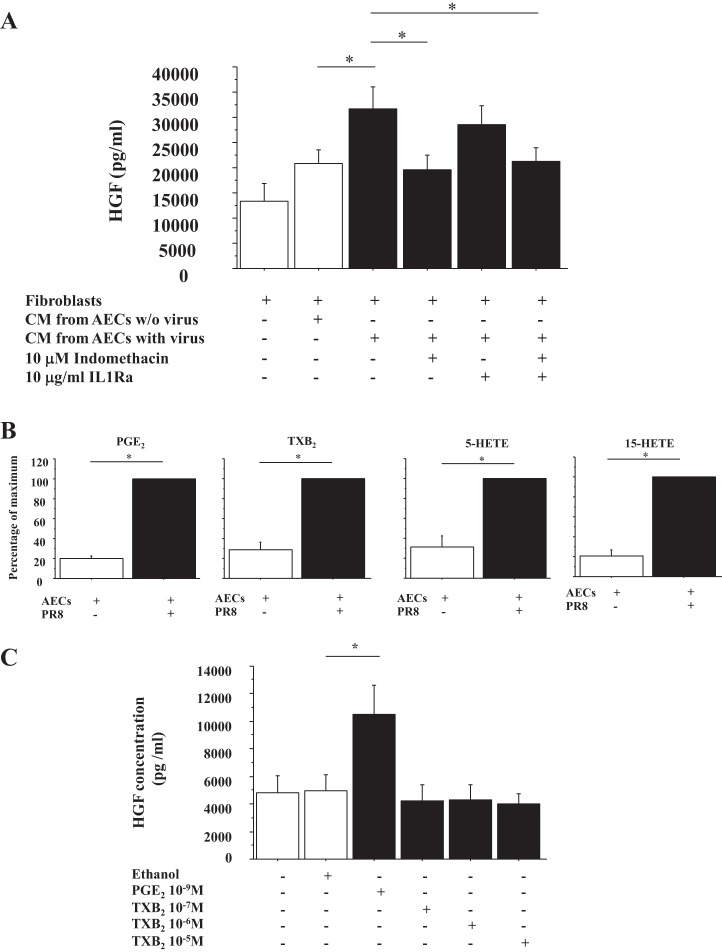
HGF secretion by FBs is enhanced by prostaglandin E_2_ (PGE_2_) secreted by influenza virus-infected AECs, which is inhibited by indomethacin. *A*: lung FBs were stimulated by conditioned medium from AECs with or without PR8 (MOI = 0.5) and with or without 10 μM indomethacin and with or without 10 μg/ml IL-1Ra, and culture medium was harvested at 48 h after stimulation. The HGF concentration of this culture medium was measured by ELISA (*n* = 3). *B*: arachidonic acid metabolites secreted by influenza virus-infected AECs were analyzed by mass spectrometry. The range of maximal stimulation: PGE_2_, 17.1–22.5 ng/sample; range of thromboxane B2 (TXB_2_), 0.26–2.16 ng/sample; range of 5-hydroxyeicosatetraenoic acid (5-HETE), 0.16–0.35 ng/sample; 15-HETE, 3.51–6.06 ng/sample; *n* = 3. *C*: lung FBs were stimulated by 10^−9^ M PGE_2_, 10^−7^ M TXB_2_, 10^−6^ M TXB_2_, or 10^−5^ M TXB_2_, and culture medium was harvested at 48 h after stimulation. The HGF concentration of this culture medium was measured by ELISA (*n* = 3). Ethanol was a vehicle control for PGE_2_ and TXB_2_.

We next evaluated factors secreted by AECs that would stimulate HGF secretion by FBs during IAV infection. Previous study using BALF from patients with ARDS showed that HGF secretion by lung FBs was stimulated by IL-1 and PGE_2_ (34). Therefore, AECs were infected by PR8 with or without indomethacin, a cyclooxygenase inhibitor, culture medium was collected at 24 h after infection, and this CM was placed on lung FBs with or without IL-1Ra. HGF secretion by FBs was stimulated by PR8 infection, which was inhibited by indomethacin but not by IL-Ra (*n* = 3) (Fig. 5*A*). To determine which eicosanoids were stimulated by PR8 infection in AECs, arachidonate metabolites in the CM were analyzed by MS (Fig. 5*B*). PR8 infection induced PGE_2_ and TXB_2_ secretion by AECs (*n* = 3) (Fig. 5*B*). Finally, to determine whether PGE_2_ and/or TXB_2_ stimulated HGF secretion by lung FBs, PGE_2_ and TXB_2_ were added to lung FBs, and the culture medium was harvested at 48 h. HGF concentrations of these media were measured by ELISA. Only PGE_2_ stimulated HGF secretion by FBs (*n* = 3) (Fig. 5*C*). MS analysis also showed that other arachidonic acid metabolites such as 5-HETE and 15-HETE were induced by influenza-infected AECs (Fig. 5*B*), whereas LTB_4_ and LTE_4_ were below the limits of quantitation.

**Fig. 6.**
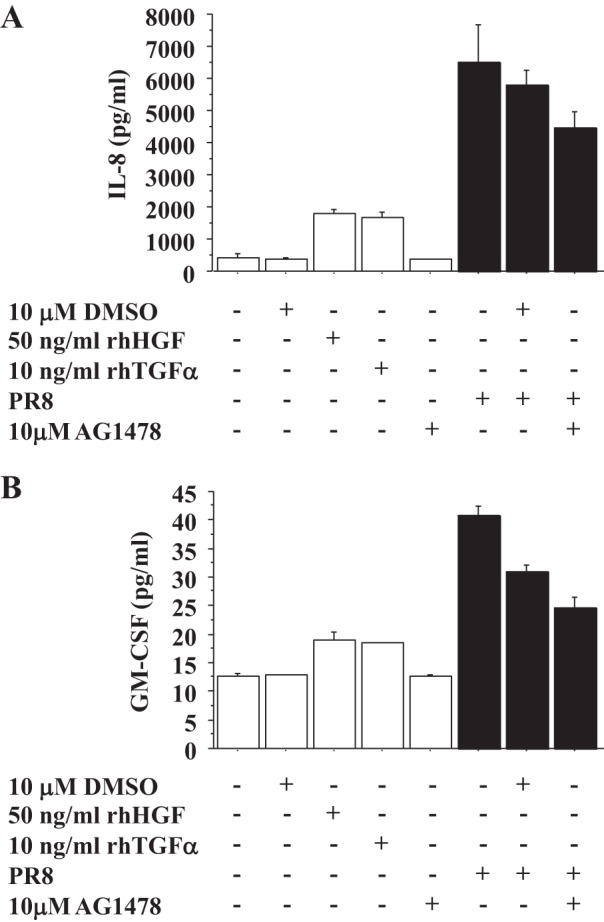
rhHGF and rhTGF-α stimulate IL-8 and GM-CSF production of alveolar type II (ATII) cells, but the basal levels of IL-8 and the level of IL-8 and GM-CSF stimulated by PR8 are not reduced by the EGFR inhibitor. *A*: concentration of IL-8 in the culture medium of ATII cells with or without rhHGF or rhTGF-α and with or without PR8 infection (MOI = 0.5) and with or without AG1478, an EGFR inhibitor, for 24 h was measured by ELISA. This is the representative data from 3 independent experiments for IL-8. DMSO was a vehicle control for AG1478. *B*: GM-CSF concentration of culture medium of ATII cells with or without rhHGF or rhTGF-α and with or without PR8 infection (MOI = 0.5) and with or without 10 μM AG1478, an EGFR inhibitor, for 24 h was measured by ELISA. This is the representative data from 3 independent experiments for GM-CSF. DMSO was a vehicle control for AG1478.

The studies above were done with AECs cultured on RTC. These cells would be differentiating toward the type I phenotype and would no longer express some of the type II markers (7, 46, 55). To study cells that maintain the type II cell phenotype, type II cells were cultured on a laminin-rich matrix in the presence of differentiation factors (33). Under these conditions, TGF-α and HGF also stimulated IL-8 and GM-CSF production (Fig. 6). However, the basal level of IL-8 and the level of IL-8 and GM-CSF stimulated by PR8 were not statistically reduced by the EGFR inhibitor AG1478.

## DISCUSSION

Influenza virus is known to induce cytokines/chemokines by primary human AECs, which regulate neutrophil accumulation into the human lungs. However, whether their induction is regulated by RTKs was not known. In this study, we identified two RTK pathways induced by IAV infection of AECs that stimulated production of the neutrophil chemoattractants IL-8 and GM-CSF. In one pathway, IAV induced TGF-α secretion that activated EGFR on AECs. The other pathway involved the stimulation of c-Met on AECs by HGF, which was produced by FBs stimulated by PGE_2_ produced by AECs infected with influenza.

IL-8 plays an important role in recruitment of neutrophils from the blood to infected or injured tissue (12). IL-8 protein production is rapidly induced by a very wide range of stimuli such as tumor necrosis factor, IL-1, bacteria, virus, and cellular stress (12). However, recent evidence suggests that airway epithelial surface signaling through EGFR is a convergent pathway producing innate immune responses to a variety of infectious and noninfectious noxious stimuli (4, 18, 21, 23, 45). Influenza virus infection of bronchial epithelial cells has been reported to shed EGFR ligands including TGF-α and activate EGFR, which in turn induces IL-8 (18, 45). Our study is the first to demonstrate that rhTGF-α induces IL-8 secretion by human AECs (Fig. 1*A*) and that influenza virus infection induces TGF-α secretion and activates EGFR, which induces IL-8 and GM-CSF secretion by human AECs (Fig. 3, *A*–*C*).

The HGF/c-Met pathway plays important roles in various injury and disease models with promoting cell survival, regeneration of tissue, and suppressing chronic inflammation and fibrosis (27). Recently, we have also shown that rhHGF and HGF secreted by lung FBs enhance wound closure in human AEC monolayers (14). Although the HGF/c-Met pathway is well recognized for its role in regeneration of AECs in humans, little is known about HGF/c-Met signaling in the innate immune response during respiratory infections including influenza infection. As far as we know, there are three reports to link c-Met signaling with IL-8 or neutrophil migration. c-Met inhibition results in reduced accumulation of neutrophils to the wound site in zebra fish (5); rhHGF stimulates the induction of CINC-1, a rat analog of IL-8, gene expression in hepatocytes (16); and c-Met signaling regulates IL-8 secretion by pancreatic cancer cell lines (11). Thus this study is the first to show that HGF/c-Met signaling regulates IL-8 and GM-CSF by human AECs, which attract neutrophils, and that influenza virus infection to AECs activates PGE_2_/HGF/c-Met pathway, which induces IL-8 secretion. As c-Met ligand, HGF, is strongly expressed by lung FBs but not by human AECs (Fig. 2), we examined the effect of HGF/c-Met signaling on IL-8 secretion by AECs during influenza infection using a coculture system of AECs with FBs. A previous study reported that HGF was increased in the BALF of patients with ARDS and that HGF secretion by FBs was induced by PGE_2_ (34). These results are similar to our IL-8 induction pathway with AECs and lung FBs during influenza infection. AECs infected with influenza secreted PGE_2_, and exogenous PGE_2_ stimulated secretion of HGF by human lung FBs. We also tested stable TXB_2_ but not the biologically active unstable precursor TXA_2_. Therefore, the IL-8 induction by HGF in our coculture system might be observed in influenza viral pneumonia in vivo.

We have also shown that GM-CSF secretion by human AECs is stimulated by rhHGF, rhTGF-α, and influenza virus through c-Met and EGFR activation. Our study is the first to demonstrate that rhHGF, rhTGF-α, and influenza virus infection induces GM-CSF secretion by human AECs through c-Met and EGFR activation. However, GM-CSF has also been reported to be induced by EGFR activation in trophoblast cells (7), keratinocytes (24), and bronchial epithelial cells (35). Another growth factor, KGF, also enhances GM-CSF secretion by murine ATII cells, and KGF enhances alveolar host defense through GM-CSF-stimulated macrophage activation in a murine bacterial pneumonia model (52). Additionally, GM-CSF is produced by murine primary ATII cells through Toll-like receptor 4 signaling, which plays a protective role on lung epithelium during Gram-negative bacterial pneumonia (40). In terms of HGF, human monocytes stimulated by rhHGF have also been shown to induce GM-CSF mRNA levels (8). These studies support our findings. In terms of roles of GM-CSF, GM-CSF simulates migration, activation, and survival of neutrophils in the sites of inflammation (19, 20, 36), whereas, in the peripheral lung, GM-CSF regulates terminal differentiation of AMs, which is critical for pulmonary surfactant homeostasis and AM-mediated innate host defense (38, 44, 50). GM-CSF is also known to protect against IAV infection and postinfluenza bacterial pneumonia by activating AMs (13, 37, 41). In addition, in the BALF of patients with ARDS, GM-CSF, G-CSF, and IL-8 are all increased in the patients compared with healthy controls, but levels of G-CSF and IL-8, but not GM-CSF, correlate with severity of pulmonary neutrophilia in ARDS (1). Therefore, although GM-CSF induces neutrophil migration, other functions of GM-CSF may be more important during influenza viral pneumonia.

Previous study shows that EGFR signaling suppresses IRF-1-dependent IP-10 (CXCL-10) and IFN-λ (IL-29) secretion in bronchial epithelial cells (18, 45) and that, in the murine influenza model, EGFR inhibition increases lymphocyte recruitment and results in decreased viral infection through IRF-1 (18, 45). However, our results are different from those reported for bronchial epithelial cells; IP-10 and IFN-λ secretion by human AECs is not altered by EGFR activation (Fig. 3*D*). In addition, although other EGFR ligands, AREG, and HB-EGF stimulate IL-8 secretion by bronchial epithelial cells (26, 30, 35) and AREG and HB-EGF are highly expressed by human AECs, in our studies, we found that AREG and HB-EGF do not induce IL-8 secretion by human AECs. Therefore, the type of EGFR ligands that stimulate IL-8 secretion and the downstream pathways regulated by EGFR activation seem to be cell and species specific.

The main and fatal complication of influenza virus infection is viral pneumonia, which can lead to ARDS. Influenza virus targets AECs, especially ATII cells, which are the first cells that influenza virus encounters after entering the alveolus. In response to viral infection, AECs produce cytokines and attract neutrophils (22, 39, 49). A key feature of ARDS is the accumulation of neutrophils in the lung, and the release of chemokines, including IL-8, from resident cells is central to neutrophil recruitment (51). Therefore, targeting EGFR and/or c-Met to suppress excessive neutrophil accumulation and subsequent inflammation may be a potential strategy for influenza virus-induced ARDS. However, there are several critical issues and limitations. First, neutrophils play an important role in innate immune defense by phagocytosing pathogens and preventing further invasion, whereas the excessive accumulation of activated neutrophils can cause unwanted bystander tissue damage. For example, removal of neutrophils in a model of influenza virus infection worsened outcomes (43). Second, c-Met and EGFR regulate multiple functional pathways such as regeneration of the damaged airway epithelium (9, 14, 28, 29, 32) and GM-CSF secretion, which might protect against influenza-induced ARDS (13, 37). For example, the EGFR inhibitor, AG1478, inhibits basal wound closure in human bronchial epithelial cells (32), and anti-HGF antibody added to mouse lung explants after influenza infection reduces alveolar epithelial regeneration (28). Therefore, in considering targeting EGFR and c-Met to suppress the excessive neutrophil accumulation and the subsequent inflammation, the potential benefits must be considered in relation to potential deleterious effects. Third, we have not investigated the relative importance of c-Met and EGFR signaling in vivo in the innate immune response to influenza virus infection and in the pathogenesis of ARDS. In a murine influenza model, EGFR inhibition protects mice from influenza infection with a decrease in infectivity by increasing leukocyte migration and IFN-λ and IP-10 secretion (18). Influenza-infected mice treated with an EGFR inhibitor have decreased levels of murine IL-8 homolog, MIP-2, and neutrophils in the lungs. The relative importance of c-Met signaling in vivo on neutrophil accumulation in influenza-infected mice has not been investigated. Determining the role of HGF in the mouse during influenza infection is, however, more complicated because murine alveolar macrophages express and secrete HGF, whereas human alveolar macrophages do not (Fig. 2). Nevertheless, recombinant HGF stimulates alveolar proliferation and alveolar repair in the mouse during influenza infection and protects against alveolar damage (28, 29).

An unexpected finding was that EGFR inhibition was less effective at reducing influenza-induced IL-8 secretion in AECs cultured to maintain the alveolar type II cell phenotype. We assume that this is because additional parallel pathways were activated in the cells that maintain the type II cell phenotype such that significant inhibition of IL-8 secretion would require inhibition of multiple pathways. However, it is also possible that TGF-α/EGFR signaling is more effective when AECs are flattened and spread. Perhaps this pathway is more dominant in type I cells and in type II cells. Resolving these issues will require isolating human type I cells and additional studies in the future. However, it also indicates that studies with bronchial epithelial cells should include studies with highly differentiated cells to compare to cells cultured on tissue culture plastic.

In summary, our study is the first to show that primary human AECs induce IL-8 and GM-CSF through c-Met and EGFR activation and that influenza infection activates c-Met and EGFR in human AECs. Although further studies with in vivo models will be necessary to determine the relative importance of c-Met and EGFR in neutrophil migration during influenza viral pneumonia, our studies using primary human AECs are an important first step in understanding the pathogenesis of influenza viral pneumonia in humans. In the future, determining the signaling pathways downstream of c-Met and EGFR responsible for cytokine/chemokine production and epithelial repair in in vivo influenza models might provide insight into potential therapeutic targets to control excessive neutrophil accumulation without dampening host defense during influenza viral pneumonia.

## GRANTS

This work was supported by grants from the National Institutes of Health (HL 106112, AI 082982, and HL 34303) and the ExxonMobil Foundation.

## DISCLOSURES

No conflicts of interest, financial or otherwise, are declared by the authors.

## AUTHOR CONTRIBUTIONS

Author contributions: Y.I., C.C.L., and R.J.M. conception and design of research; Y.I., K.C., R.L.Z., and R.C.M. performed experiments; Y.I., K.C., R.L.Z., and R.C.M. analyzed data; Y.I., R.L.Z., C.C.L., R.C.M., and R.J.M. interpreted results of experiments; Y.I. prepared figures; Y.I. drafted manuscript; Y.I., R.L.Z., C.C.L., and R.J.M. edited and revised manuscript; Y.I., K.C., R.L.Z., C.C.L., R.C.M., and R.J.M. approved final version of manuscript.
